# Exploring the Synergy between PARP and CHK1 Inhibition in Matched *BRCA2* Mutant and Corrected Cells

**DOI:** 10.3390/cancers12040878

**Published:** 2020-04-04

**Authors:** Hannah L Smith, Lisa Prendergast, Nicola J Curtin

**Affiliations:** 1 Newcastle Centre for Cancer, Translational and Clinical Research Institute, Faculty of Medical Sciences, Newcastle University, Newcastle upon Tyne NE2 4HH, UK; hannah.smith2@newcastle.ac.uk; 2 Cancer Research UK Drug Discovery Unit, Newcastle Centre for Cancer, Translational and Clinical Research Institute, Faculty of Medical Sciences, Newcastle University, Newcastle upon Tyne NE2 4HH, UK; Lisa.Prendergast@newcastle.ac.uk

**Keywords:** PARP, ATR, CHK1, replication stress, homologous recombination DNA repair, cell cycle, cytotoxicity

## Abstract

PARP inhibition results in the accumulation of DNA SSBs, causing replication stress (RS) and lesions that can only be resolved by homologous recombination repair (HRR). Defects in HRR, e.g., due to *BRCA2* mutation, confer profound sensitivity to PARP inhibitor (PARPi) cytotoxicity. In response to RS, CHK1 is activated to signal to S and G2/M cell cycle checkpoints and also to HRR. To determine the relative contribution of these two functions of CHK1 to survival following PARPi exposure, we investigated the effects of rucaparib (a PARPi) and PF-477736 (a CHK1 inhibitor) alone and in combination in cells with mutated and corrected *BRCA2*. The *BRCA2* mutated V-C8 cells were 1000× more sensitive to rucaparib cytotoxicity than their matched *BRCA2* corrected V-C8.B2 cells, but no more sensitive to PF-477736 despite having seven-fold higher levels of RS. PF-477736 caused a five-fold enhancement of rucaparib cytotoxicity in the V-C8.B2 cells, but no enhancement in the V-C8 cells. This differential sensitivity was not due to a difference in PARP1 or CHK1 expression or activity. PF-477736 increased rucaparib-induced RS (γH2AX foci) and completely inhibited RAD51 focus formation, indicating a profound suppression of HRR. Our data suggested that inhibition of HRR was the main mechanism of sensitisation to rucaparib, compounded with an inhibition of cell cycle checkpoints by PF-477736.

## 1. Introduction

Replication stress (RS) is a key source of genomic instability, an enabling characteristic of cancer [1]. RS is increased in cancer due to an almost ubiquitous loss of G1 checkpoint control [2] and unrepaired DNA lesions encountering the replication fork. Poly(ADP-ribose) polymerase (PARP) is a key component of the DNA damage response by promoting DNA base excision repair/single-strand break repair (BER/SSBR) [3]. PARP is the first line of defence against the most common type of endogenous DNA damage, oxidative stress, a major contributor to RS. PARP inhibitors (PARPi) are a significant breakthrough in the treatment of cancer by exploiting cancer-specific defects in homologous recombination DNA repair (HRR), e.g., due to *BRCA* mutations. Toxicities associated with these drugs are generally mild [4]. Three PARPi are currently approved for the treatment of ovarian cancer, the success being largely due to the high frequency (>50%) of HRR defects in this cancer type [5,6,7].

The high levels of RS and loss of G1 control make cancer cells dependent on S and G2/M cell cycle checkpoint control [8]. Checkpoint kinase 1 (CHK1) is a pivotal checkpoint kinase signalling RS to cell cycle arrest through inactivation of cdc25A and cdc25C. Cdc25A and cdc25C are phosphatases that remove inactivating phosphates on CDK2 and CDK1, respectively. Since CDK2 is required for S-phase entry and progression and CDK1 is needed for mitosis, activation of CHK1 leads to S and G2/M arrest. CHK1 has also been shown to phosphorylate RAD51 and thus has key involvement in signalling to HRR, as well as halting the cell cycle to allow repair to occur [9,10]. 

CHK1 inhibitors have the potential to counteract HRR-mediated PARPi resistance [11]. Indeed, PARPi and CHK1 inhibitors have been shown to interact to cause increased cytotoxicity in breast and ovarian cancer cells, which was mediated by inhibition of HRR and increased DNA damage [12,13]. However, to date, no investigations have been carried out in paired HRR competent and HRR defective (HRD) cell lines to confirm this as the mechanism. 

To better understand the mechanisms underlying the synergy between PARP and CHK1 inhibitors, we used paired *BRCA2* mutant (V-C8) and corrected (V-C8.B2) cells. We examined the effects of the clinically approved PARPi, rucaparib, and the CHK1 inhibitor, PF-477736, that has undergone clinical evaluation (NCT00437203) on target enzyme activity and inhibition, cell cycle control, DNA repair, and cytotoxicity. Our data suggest that CHK1 inhibition results primarily in an HRD phenotype, and this is synthetically lethal with PARP inhibition. 

## 2. Results

### 2.1. V-C8 Cells Are More Sensitive to Rucaparib, but not PF-477736, and PF-477736 only Sensitised V-C8.B2 Cells to Rucaparib

**Figure 1:**
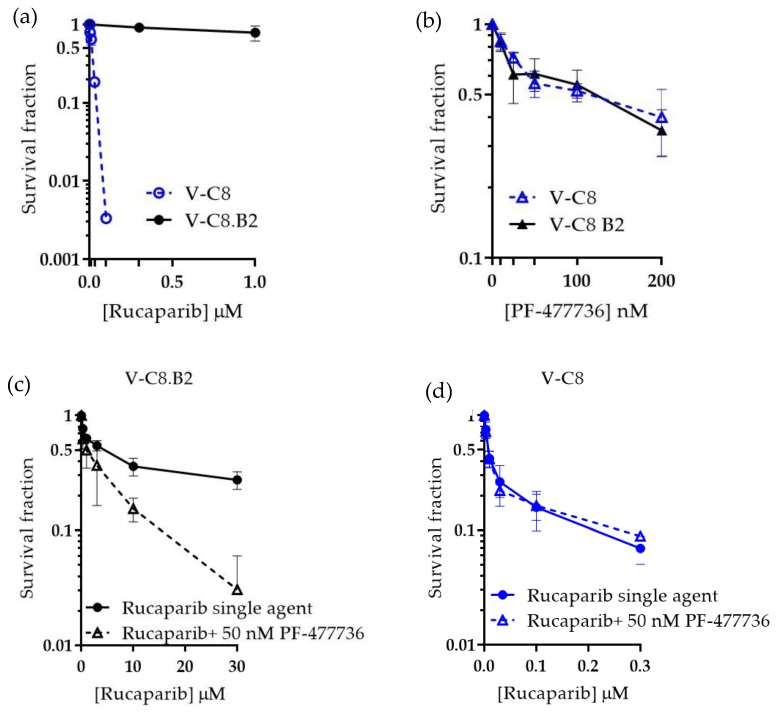
Differential cytotoxicity and synergy of rucaparib and PF-477736 in *BRCA2* mutant and corrected cells. V-C8 and V-C8.B2 cells were exposed to drugs at the indicated concentration for 24 h prior to replacement with drug-free medium for 7–10 days to allow colony formation. (**a**) Rucaparib, (**b**) PF-477736, (**c**) the combination of rucaparib with 50 nM PF-477736 in V-C8 B2 cells, and (**d**) the combination of rucaparib with 50 nM PF-477736 in V-C8 cells. Data are the mean and standard error of three independent experiments.

Colony formation assays were used to determine the potency of rucaparib across V-C8 and V-C8.B2 cell lines. As expected, the HRD V-C8 cells were particularly sensitive to rucaparib (LC_50_ < 0.01 µM) and significantly more sensitive compared to matched HRR-competent V-C8.B2 cells (LC_50_ > 10 μM, *p* < 0.001) (Figure 1a). In contrast, no significant difference in cytotoxicity to PF-477736 (Figure 1b) was observed between the cell lines as both V-C8 and V-C8.B2 cells had similar LC_50_ (100.9 and 87.5 nM, respectively). This suggested that *BRCA2*/HRD status was not a determinant of sensitivity to CHK1 inhibitor PF-477736. Survival in V-C8 and V-C8.B2 cell was inhibited by less than 50% (44.0 ± 5.9 and 41.9 ± 3.5 %, respectively) at 50 nM PF-477736, so this concentration was used for further studies. 

We next tested whether the CHK1 inhibitor could potentiate PARPi in HRR competent and defective cells. The survival of cells was evaluated when exposed to a range of rucaparib concentrations (V-C8, 0–0.3 µM, V-C8.B2, 0–30 µM, to account for increased sensitivity to rucaparib) with or without 50 nM PF-477736. In V-C8.B2 cells, co-incubation with PF-477736 decreased the LC_50_ of rucaparib 4.8-fold ± 2.7 (Figure 1c). PF-477736 did not sensitise HRD V-C8 cells to rucaparib (Figure 1d). This differential sensitisation of HRR functional and dysfunctional cells suggested PF-77736 was sensitising HRR competent cells by inhibiting HRR. 

### 2.2. The Difference in Rucaparib Sensitivity Is not Due To Differential PARP-1 Expression between Cell Lines

**Figure 2:**
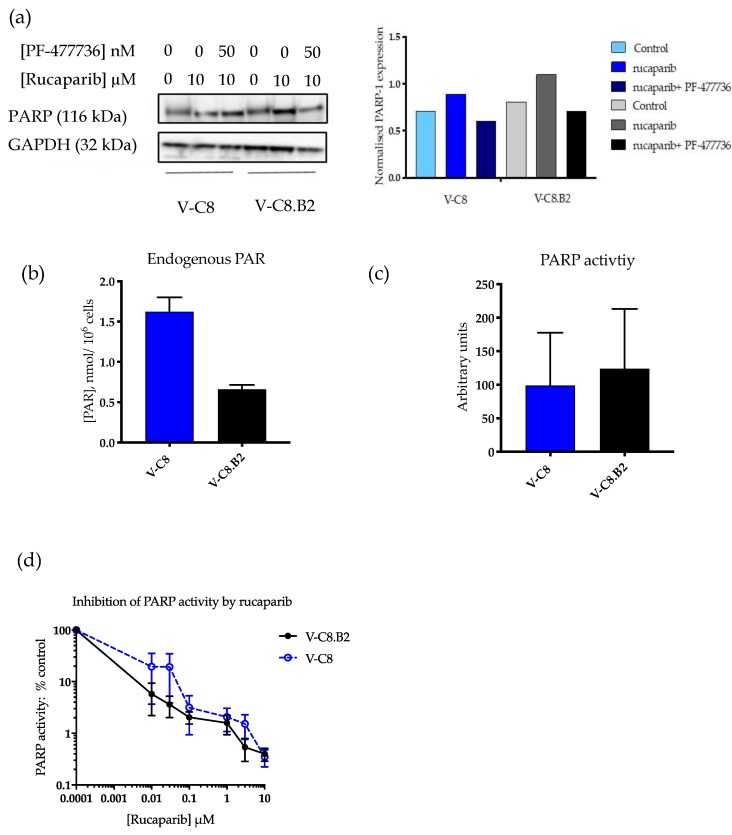
PARP-1 expression, activity, and inhibition by rucaparib in *BRCA2* mutant and corrected cells. (**a**) V-C8 and V-C8.B2 cells were exposed to drugs at the indicated concentration for 24 h prior to Western blot. Chemiluminescence was quantified using Syngene software. Bar charts are PARP-1 expression relative to the GAPDH loading control. Data are from a single representative experiment. (**b**) Endogenous PAR was measured in the absence of oligonucleotide and NAD+ by reference to a standard curve. Data are the mean and standard deviation of three independent experiments. (**c**) PARP activity (mean pixel values) was measured in the presence of oligonucleotide and NAD+. Data are the mean and standard deviation of three individual experiments. (**d**) PARP activity following exposure to increasing concentrations of rucaparib. Data are the mean and standard error of three individual experiments.

To exclude differences in PARP1 expression, activity, or inhibition as factors contributing to the differential sensitisation to rucaparib by PF-477736, we measured PARP expression and activity in both cell lines. PARP1 levels were not substantially different between cell lines and were only modestly affected by rucaparib and PF-477736 (Figure 2a and Figure S1). Endogenous PAR levels were 2.3-fold higher in the HRD V-C8 cells compared to the V-C8.B2 cells (Figure 2b), possibly reflecting a higher level of endogenous DNA breakage activating PARP. There was no significant difference between the baseline PARP activity of both cell lines (Figure 2c). Rucaparib inhibited PARP activity to a similar extent in both V-C8 and V-C8.B2 cells with IC_50_ values of 59 and 53 nM, respectively (Figure 2d). In both cell lines, a concentration of 0.1 µM rucaparib inhibited PARP activity >95%. This concentration only killed 3% of V-C8.B2 cells compared with >99% of V-C8 cells, demonstrating that the differential cytotoxicity was determined by HRR status rather than the extent of PARP inhibition.

### 2.3. Rucaparib Activates CHK1 Similarly in Both Cell Lines, and This Activation Is Inhibited by PF-477736

**Figure 3:**
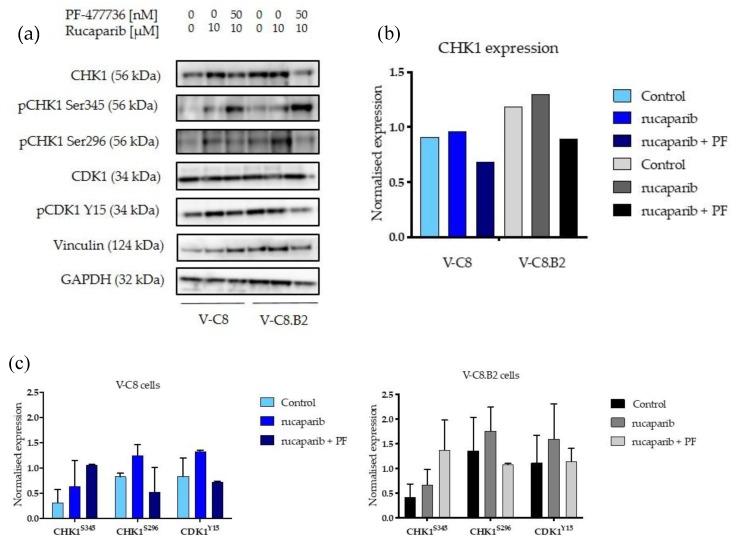
CHK1 expression, activation by rucaparib, and inhibition by PF-477736 in *BRCA2* mutant and corrected cells. Cells were exposed for 24 h to DMSO, rucaparib, or rucaparib and PF-477736 and analysed for pCHK^S345^, CHK1, and pCDK1^Tyr15^. Vinculin was used as a loading control for pCHK^S345^, CHK1, pCDK1^Tyr15^ and CDK1, and GAPDH was used as the loading control for CHK1^S296^ (**a**) Western blot from a single representative experiment. (**b**) Densitometry was calculated using Syngene software of CHK1 expression normalised to Vinculin. (**c**) Normalised densitometry of phosphorylated CHK1 and CDK1. Data are the mean and standard deviation of three individual experiment.

Another possible explanation for the differential sensitisation by PF-477736 could have been a difference in the activation of CHK1 by rucaparib and a difference in the extent to which it was inhibited by PF-77736. The expression of CHK1 in the native and phosphorylated forms and downstream signalling to CDK1 were determined by Western blot (Figure 3a and Figure S2). CHK1 expression was slightly lower in V-C8 cells compared to V-C8.B2 cells, but this was not significant (Figure 3b). Rucaparib (10 µM) activated checkpoint signalling through ATR and CHK1 in both cell lines (Figure 3a). CHK1^S345^ levels (indicating ATR activation) were increased 2.1-fold and 1.6-fold by rucaparib in V-C8 and V-C8.B2 cells, respectively. CHK1 activity (CHK1^S296^) was increased by rucaparib to a similar extent in V-C8 and V-C8.B2 cells (1.5-fold and 1.6-fold, respectively) (Figure 3c). In both cell lines, PF-477736 (50 nM) inhibited this activation of CHK1, by 91.4% ± 12.2 in V-C8 cells and 75.6% ± 17.6 in V-C8.B2 cells. The difference in inhibition of CHK1^S296^ by PF-477736 was not statistically significantly different between the two cell lines, suggesting this did not contribute towards the difference observed in sensitisation. Downstream of CHK1, PF-477736 similarly inhibited phosphorylation of CDK1 (CDK1^Y15^) by 69.6% ± 24.8 in V-C8 cells and 56.88 % ± 26.1 in V-C8.B2 cells. Upstream of CHK1, PF-477736 activated ATR 2.1-fold ± 1.3 in V-C8 cells and 2.4-fold ± 1.2 in V-C8.B2 cells, suggesting an increased reliance on ATR when CHK1 was inhibited.

### 2.4. Rucaparib Causes S-phase and G2 Accumulation, Which Is Attenuated by PF-477736

**Figure 4:**
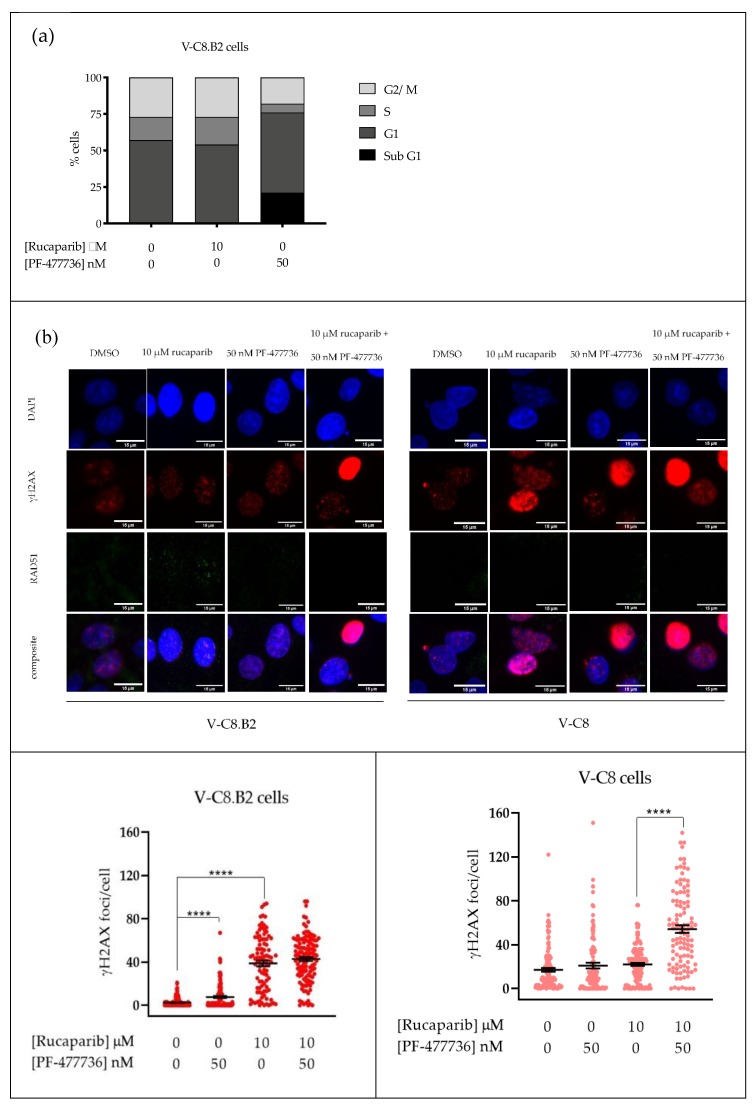

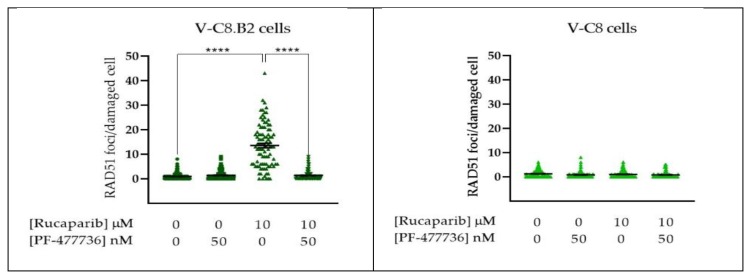
Impact of PF-477736 on cell cycle and homologous recombination repair (HRR) in rucaparib-treated cells. Cells were exposed for 24 h to DMSO, rucaparib, or rucaparib with PF-477736 at the indicated concentrations. (**a**) Cell cycle analysis of V-C8.B2 cells; data are from a single experiment. (**b**) Representative images are shown above quantified γH2AX and RAD51 foci, where each data point represents a single nucleus. RAD51 foci were only counted in cells with >mean γH2AX foci in untreated cells. Data are pooled from three independent experiments.

The effect of rucaparib and PF-477736 on the cell cycle was analysed to assess if the mechanism of sensitisation was by cell cycle checkpoint signalling. In V-C8.B2 cells, rucaparib caused a modest increase in S-phase (19.31%). Co-exposure to PF-477736 reduced the S-phase by 64.5% and G2/M by 33.6% compared to control cells, and this was accompanied by an increase in the sub-G1 fraction of 21.5%, suggesting that cells were progressing through S and G2/M with damaged DNA and dying, probably by mitotic catastrophe (Figure 4a). 

### 2.5. Rucaparib Inhibition Leads to Increased DNA Damage and HRR, and PF-477736 Inhibits HRR

The induction of replication stress by rucaparib, resulting in collapsed replication forks following accumulation of endogenous DNA damage, is commonly measured by γH2AX phosphorylation. At baseline, untreated V-C8 cells had on average seven-fold more γH2AX foci/cell, compared to V-C8.B2 cells, reflecting their inability to resolve RS through HRR (Figure 4b). In V-C8.B2 cells, rucaparib increased γH2AX foci/cell 16-fold (*p* < 0.0001), PF-477736 caused a three-fold increase in γH2AX foci/cell (*p* < 0.0001), and the γH2AX foci/cell following the combination were higher still, but not significantly different from rucaparib alone. 

In V-C8 cells, the already high levels of γH2AX foci were only increased by 20% by PF-477736 and 30% by rucaparib (Figure 4b, not significant). The combination increased the number of foci approximately three-fold (*p* < 0.0001). Due to the *BRCA2* mutation in these cells, they had very low levels of RAD51 foci, which were not increased by rucaparib or PF-477736 alone or in combination.

In marked contrast, in parallel with the induction of DNA breaks (γ-H2AX) in V-C8.B2 cells, rucaparib exposure caused an approximately 13-fold increase (*p* < 0.0001) in RAD51 foci in γ-H2AX-positive cells. Importantly, PF-477736 completely abrogated the rucaparib induction of RAD51 (significantly different from rucaparib alone, *p* < 0.0001), and the levels of foci were similar to control V-C8.B2 and the HRD V-C8 cells. These data indicated that the inhibition of CHK1 totally abolished HRR and led to a greater accumulation of collapsed replication forks.

## 3. Discussion

Defects in either *BRCA1* or *BRCA2* have been known to result in a profound sensitivity to PARP inhibition since 2005 [14,15]. Rucaparib has previously been documented to be ≥1000× more cytotoxic to *BRCA2* mutant V-C8 cells compared to their matched *BRCA2* corrected V-C8.B2 cells (Patent WO/2005/012305), which we confirmed here. Interestingly, *BRCA2* was not a determinant of PF-477736 sensitivity. RS was thought to be a determinant of sensitivity to cell cycle checkpoint inhibitors, so this was somewhat surprising given that untreated V-C8 cell had seven-times more H2AX foci than V-C8.B2 cells. In previous studies, V-C8 cells were more sensitive to the ATR inhibitor VE-821 [16], possibly RS being a greater determinant of sensitivity to ATR than CHK1 inhibition. 

Here, we confirmed that the observed difference in rucaparib sensitivity was not due to different PARP-1 expression levels or activity between the cell lines. This contrasts with previous reports of substantially higher levels of PARP activity in V-C8 compared to V-C8.B2 cells [17]. This may reflect differences in the method of detecting PARP activity. In our study, we used a GCLP-validated assay that has been used as a pharmacodynamic biomarker in PARPi clinical trials [18]. However, like this previous study, endogenous PAR levels were around two-fold higher in *BRCA2* mutant cells than wildtype, possibly indicating higher levels of endogenous DNA damage. In support of this hypothesis, there was approximately seven-times as much γH2AX foci formation in untreated *BRCA2* mutant V-C8 cells compared to *BRCA2* corrected V-C8.B2. The differential rucaparib sensitivity observed in cytotoxicity experiments was clearly due to *BRCA2* mutation and the synthetically lethal relationship between BER and HRR pathways. Therefore, it is interesting that PF-477736 only sensitised V-C8.B2 cells and not V-C8 cells to rucaparib, suggesting the CHK1 inhibitor was primarily acting on HRR. 

We demonstrated that rucaparib induced RS to a higher extent in V-C8.B2 cells; however, this was most likely due to the high levels of RS in V-C8 control cells. CHK1 was activated to a similar extent by rucaparib in both V-C8 and V-C8.B2 cells, and PF-477736 caused a similar inhibition in both cell lines, so the differential sensitisation observed was not due to a greater activation or inhibition of the pathway. Rucaparib caused a similar increase in pCDK1^Y15^, which was inhibited to a similar degree by PF-47736 in both cell lines, indicating that the downstream checkpoint signalling was intact and equally responsive to the two inhibitors in both cell lines. Therefore, this was not a factor in the differential sensitisation of VC8.B2 cells. Increased CHK^S345^ phosphorylation in both V-C8 and V-C8.B2 cells was also observed in response to CHK1 inhibition with PF-477736, suggesting upstream ATR activation as previously described [19,20].

Cell cycle analysis showed only a modest S-phase accumulation caused by rucaparib, probably associated with the increase in RS that was demonstrated by the increase in γH2AX (Figure 4). PF-477736 not only abolished this rucaparib-induced S-phase arrest, but also reduced both S- and G2-phases relative to untreated controls. This reduction in S and G2 was accompanied with a corresponding increase in the sub-G1 fraction, suggesting damaged cells were forced into mitosis before they could undertake repair, as has been reported for other PARP and CHK1 inhibitor combinations [21,22].

Although these data indicated that inhibition of cell cycle checkpoints by PF-477736 made a contribution to the cytotoxicity of rucaparib, this is unlikely to explain the differential sensitisation of the *BRCA2* corrected compared to the *BRCA2* mutant cells. We therefore tested the hypothesis that the primary mechanism of sensitisation was via PF-477736-mediated inhibition of HRR. As predicted, PF-477736 profoundly inhibited HRR, completely abolishing RAD51 foci formation. In a study of breast cancer cell lines, prexasertib synergised with the PARPi olaparib by causing S-phase arrest and inhibiting HRR/RAD51 foci [23]. In contrast, in high-grade serous *BRCA*-wildtype ovarian cancer cells, prexasertib synergised with olaparib by inhibiting G2/M arrest, as well as also inhibiting olaparib-induced RAD51 foci formation [13]. Clearly, the various molecular pathologies of these cells complicate the interpretation of the data.

## 4. Materials and Methods

### 4.1. Chemicals and Reagents

All chemicals and reagents used were obtained from Sigma-Aldrich, unless stated otherwise. Rucaparib was gifted from Pfizer Global R&D. Both rucaparib and PF-477736 (Selleckchem, Houston, TX, USA) were dissolved in dry DMSO at respective concentrations of 20 mM and 5 mM and stored at −80 °C before use. 

### 4.2. Cell Culture

VC-8 and VC-8.B2 cells, a gift from Malgorzata Zdzienica, Leiden University [24], were maintained in exponential growth phase (<70–80% confluence) in DMEM with 10% foetal bovine serum (FBS; Thermo Fisher Scientific, Waltham, MA, USA) and incubated at 37 °C, 5% CO_2_, and 95% humidity. VC8.B2 cells contained a BAC containing the *BRCA2* gene and maintained under selection with 200 µg/mL G-418 (Thermo Fisher Scientific, Waltham, MA, USA). Cells were mycoplasma free. 

### 4.3. Cytotoxicity Assay

Exponentially growing cells were seeded at various densities in 6 well plates, allowing 3 different densities for each drug concentration estimated to give 20–200 colonies following drug treatment. After attachment, cells were exposed to various concentrations of rucaparib or PF-477736 alone or the combination of rucaparib ± 50 nM PF-477736 in DMSO or DMSO alone at a final concentration of 0.5% for 24 h. The medium was replaced with fresh medium and the cells left to incubate for 7–10 days to form colonies. The colonies were fixed in methanol:acetic acid (3:1) stained with 0.4% crystal violet, and colonies of >30 cells were counted by eye. Graphs were plotted using Graphpad Prism 6 software (San Diego, CA, USA). 

### 4.4. Measurement of PARP Activity and Inhibition

Following incubation with rucaparib (0–10 µM) for 30 min, PARP activity was measured in permeabilised cells at 26 °C in the presence of 350 nM NAD+ and 10 mg/mL 12 mer palindromic oligonucleotide to activate the enzyme, as described previously [25,26]. Following transfer to a nitrocellulose membrane (GE Healthcare Life, Sciences, Amersham, Buckinghamshire, UK) the product, PAR, was measured with 10H anti-PAR monoclonal antibody (Enzo life sciences, Exeter, UK) overnight at 4 °C and HRP-conjugated goat anti-mouse secondary antibody (Dako, Santa Clara, CA, USA) diluted 1:1000 in PBS-Tween (PBS-T) 5%. Clarity Max ECL Western substrate (Bio-Rad, Hercules, CA, USA) was added to the membrane, chemiluminescence imaged using the GBox and Genesys software (Syngene, Cambridge, UK), and quantified with reference to a standard curve of 0–25 pmol purified PAR (Enzo life sciences, Farmingdale, NY, USA) after subtraction of background reactions in the absence of NAD and oligonucleotide. Baseline PAR levels were measured using 50–100-times the cells used for inhibition experiments and with no oligonucleotide or NAD+ present. Purified PAR standard was diluted accordingly to a concentration of 0–25 pmol. 

### 4.5. Measurement of CHK1 Activation and Inhibition by Western Blot

Exponentially growing cells in 100 mm dishes were exposed to media containing DMSO, 10 μM rucaparib, or 10 μM rucaparib and 50 nM PF-477736 for 24 h before extraction with 250 µL per dish of phosphosafe extraction reagent with protease cocktail inhibitor (Thermo Fisher Scientific, Waltham, MA, USA) at a 1:100 dilution per dish at 4 °C for 5–7 min, then scraped on ice into Eppendorf tubes. Following centrifugation at 8000× *g* for 10 min at 4 °C, the protein concentration of the supernatants was measured using a BCA protein assay kit (Thermo Fisher Scientific, Waltham, MA, USA) and diluted with deionised water to 0.5–1 mg/mL. XT sample buffer (Bio-Rad, Hercules, CA, USA) and XT reducing agent (Bio-Rad, Hercules, CA, USA) were added at constants of 25% and 0.5%, respectively. Samples were heated at 90 °C for 5 min, then 30 µg were loaded/well of 3–8% Criterion XT tris-acetate gels (Bio-Rad, Hercules, CA, USA), and the gel ran at 150V for approximately 1 h alongside HiMark pre-stained protein standard (Thermo Fisher Scientific, Waltham, MA, USA) in diluted 20× XT tricine with deionised water. The separated proteins were transferred onto a nitrocellulose membrane (Amersham, Buckinghamshire, United Kingdom) at 100 V for 1 h. The membrane was blocked for 1 h at room temperature in 5% milk in TBS-tween (TBST). Primary antibodies PARP-1 (#ab227244 Abcam, Cambridge, UK), vinculin (#4650), GAPDH (14C10 #2118), phospho-CHK1 (Ser296) (D3O9F #90178), phospho-Chk1 (Ser345) (#2341), and phospho-CDK1 (Tyr15) (10A11 #4539) were each diluted 1:1000 in TBS-Tween (TBS-T) and 5% bovine serum albumin (BSA) and left to incubate overnight at 4 °C (all obtained from Cell signalling, Massachusetts, United States except PARP-1). The membrane was cut into sections (approximately 30–45 kDa, 45–70 kDa, and 70–220 kDa), washed in TBS-T, before adding anti-rabbit goat polyclonal HRP secondary antibody (Cell Signaling Technology, Danvers, MA, USA) diluted 1:2000 in 5% skimmed milk in TBST at room temperature for 1 h. After washing with TBST, Clarity Max ECL Western substrate (Bio-Rad, Hercules, CA, USA) was applied to the membrane slices and chemiluminescence measured using Syngene software on the G-box. 

### 4.6. Cell Cycle Analysis

Exponentially growing cells were exposed to DMSO, 10 μM rucaparib or 10 μM rucaparib with 50 nM PF-477736 for 24 h. Cells were washed twice with PBS, with each washing collected to ensure no loss of cells, before being trypsinised and harvested. Following centrifugation at 1500 rpm for 5 min, the supernatant was discarded, and the remaining cell pellet was resuspended in 1 mL ice cold PBS and centrifuged (3000 rpm, 5 min). The supernatant was removed, and 1 mL of 70% ethanol was added dropwise to the cell pellet. Samples remained at 4 °C for a minimum of 1 h. Prior to staining, cells were washed twice in PBS to remove ethanol before eventually being resuspended in 700 μL PBS. RNase was added to cells (12 µL of 1 mg/mL stock) alongside propidium iodide stain (20 μL of 1mg/mL stock). Cells were incubated in dark conditions at 37 °C for a minimum of 30 min. Analysis was carried out using Attune Nxt Cytometer. De Novo FCS Express 7 software was used to analyse the data.

### 4.7. Homologous Recombination Repair Assay

Cells were seeded at 0.5 × 10^5^ cells/mL onto coverslips and incubated for 24 h before being exposed to either DMSO, 50 nM PF-477736, 10 μM rucaparib, or 10 μM rucaparib with 50 nM PF-477736 for 24 h. Cells were then washed with PBS and fixed with ice cold methanol for a minimum of 1 h at −20 °C. Coverslips were then washed with PBS 0.2% Triton-X-100 and blocked (2% BSA, 10% skimmed milk powder (Marvel, UK), 10% goat serum) for 1 h at room temperature before being incubated with anti-RAD51 (Abcam, Cambridge, UK) diluted 1:500 in blocking buffer at 4 °C overnight. After washing in PBS, 0.2% Triton X-100 anti-phospho-histone H2AX (Merck, Branchburg, NJ, USA) was added to coverslips at 1:1000 and incubated for 1 h. After washing, secondary antibodies, Alexa Fluor 488 and Alexa Fluor 546 (ThermoFisher Scientific, Waltham, MA, USA), were added and incubated for 1 h, in dark conditions. Coverslips were washed then exposed to 0.5 µg/mL DAPI solution. Coverslips were mounted onto microscope slides using H-1400 hard set mounting media (Vector Laboratories, Burlingame, CA, USA). Slides were stored in dark conditions and left to dry prior to imaging. Cells were imaged using the Leica DM6 LED fluorescence microscope (Leica microsystems GmbH, Wetzlar, Germany). Images were analysed using Fiji ImageJ software [27]. In DMSO-treated cells, the mean γH2AX foci were calculated. Cells (control and treated) with more than the mean number of γH2AX foci in control cells were deemed γH2AX positive. RAD51 foci were only counted in γH2AX positive cells. 

## 5. Conclusions

**Figure 5:**
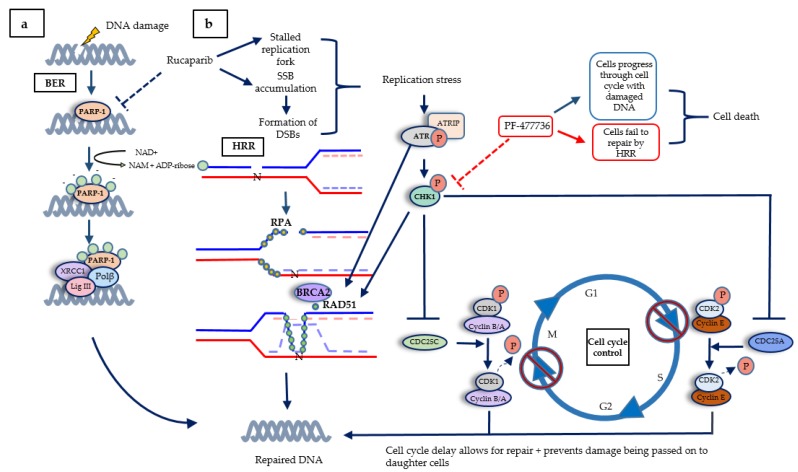
The DNA damage response, highlighting cross-talk between the cell cycle and DNA repair. (**a**) Base excision repair (BER) is the first line of defence when DNA damage occurs. PARP activation by SSB recruits XRCC1, Pol β, and Lig III to repair the DNA. (**b**) When PARP is inhibited, SSB accumulate, causing stalled replication forks, and single-ended DSBs, causing fork collapse. BRCA2 facilitates replacing RPA with repair protein RAD51, which subsequently forms a filament to search for the specific homologous sequence on the sister chromatid as a template for repair and re-start. PARPi-induced replication stress activates ATR, which initiates a signalling cascade. ATR activates CHK1 by phosphorylation at serine 345, causing CHK1 to autophosphorylate at serine 296, to achieve full activation. CHK1 inactivates CDC25A/C, thereby preventing the removal of inhibitory phosphorylation on CDK2 and 1, respectively, thus preventing S-phase progression and mitosis. The pathway also promotes HRR as CHK1 activates BRCA2 and RAD51 by phosphorylation, and ATR also phosphorylates RAD51.

On the basis of our data in matched cell lines that differed only in their BRCA2 status, we proposed the model shown in Figure 5. That is, the RS caused by the accumulation of unrepaired SSB when PARP was inhibited led to collapse of replication forks. These lesions triggered activation of the ATR- CHK1 pathway that signals to cell cycle arrest, but the major contribution that CHK1 makes was to protect cells from PARPi cytotoxicity by promoting HRR. The use of a CHK1 inhibitor may therefore be useful not only for tumours that are intrinsically PARPi resistant due to functional HRR, but may also overcome acquired resistance to PARPi in BRCA mutant tumours that have restored HRR function. Recent studies with ovarian cancer PDX models demonstrated that the combination of the CHK1 inhibitor prexasertib with olaparib caused greater tumour growth delay and survival in both olaparib sensitive and resistant tumours [28]. Our data along with this PDX study may help in the interpretation of the results of clinical trials of PARPi-CHK1 inhibitor combinations, such as NCT03057145, in which olaparib is being investigated in combination with prexasertib. 

## 6. Patents

Helleday T and Curtin NJ. Therapeutic Compounds (PARP inhibitors in homologous repair/BRCA defective cancer) Patent Application Number PCT/GB2004/003183. Publication number WO 2005/012305 A2 Divisional application 16th April 2004 GB 0408524. WO2005012305A3. 

## Supplementary Materials

The following are available online at https://www.mdpi.com/2072-6694/12/4/878/s1: Figure S1: PARP1 expression in V-C8 and V-C8.B2 cells, Figure S2: CHK1 expression, activation by rucaaprib and inhibition by PF-477736 in V-C8 and V-C8.B2 cells.
