# Supplementary figures and images for: Exploring the Synergy between PARP and CHK1 Inhibition in Matched BRCA2 Mutant and Corrected Cells
Source: Cancers (Basel). 2020 Apr 4;12(4):878. doi: 10.3390/cancers12040878 (PMC7226483; doi:10.3390/cancers12040878)

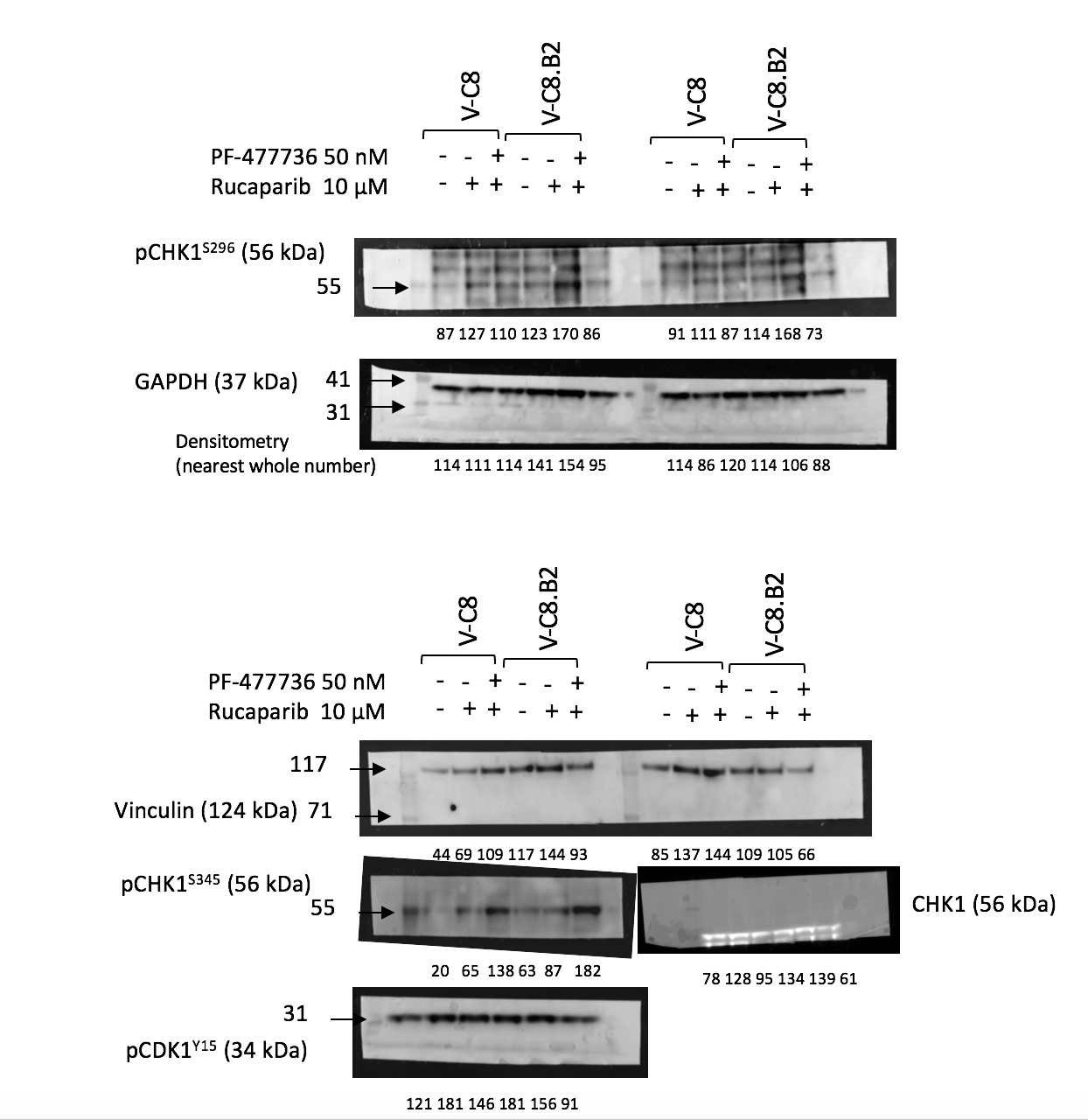

Supplement: Supplementary file 1 [file cancers-12-00878-s001.zip › Cancer supplementary/Figure S2.tiff]

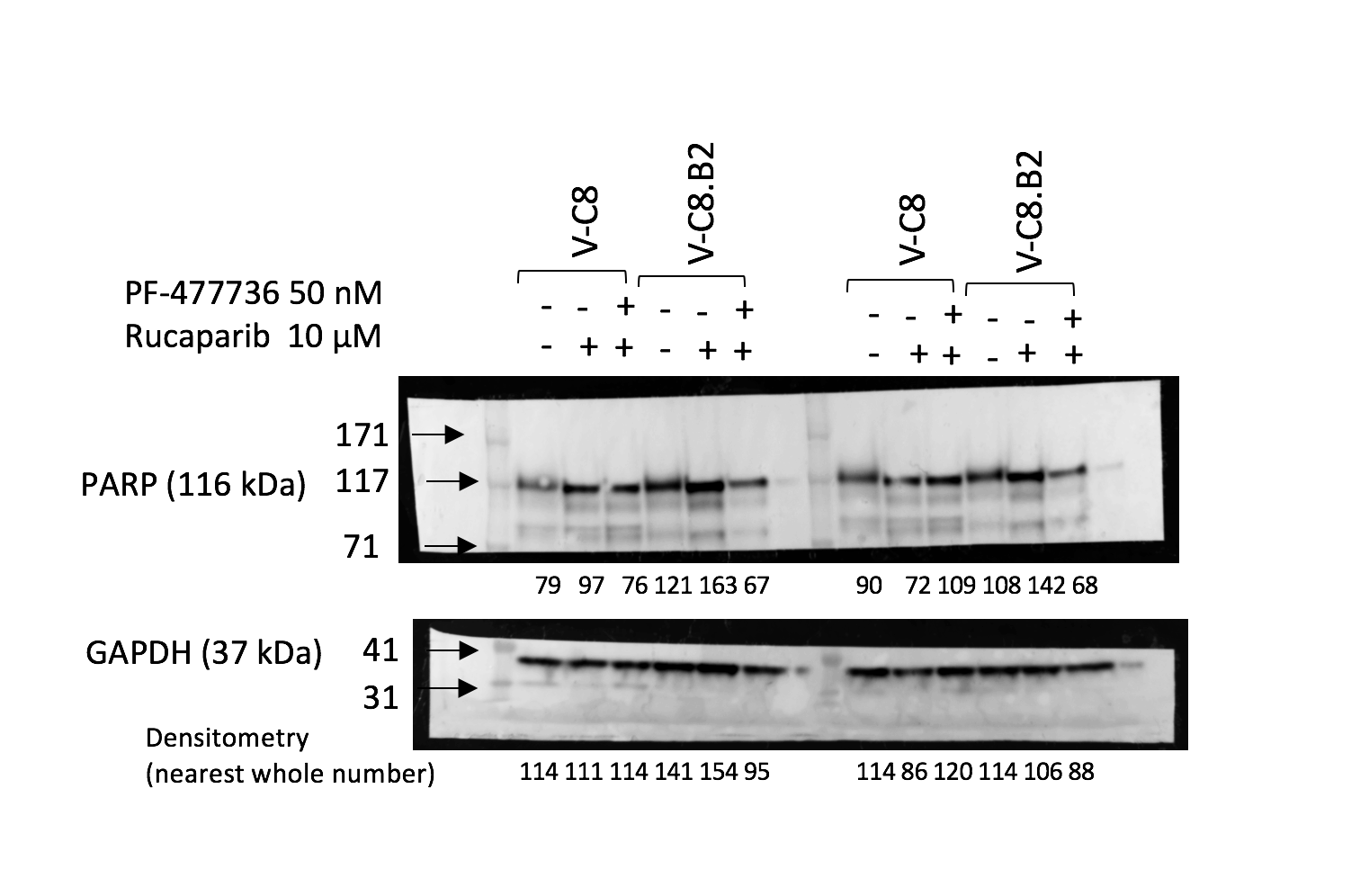

Supplement: Supplementary file 1 [file cancers-12-00878-s001.zip › Cancer supplementary/Figure S1.tiff]
